# Supplementary material for: Lipid Remodeling Confers Osmotic Stress Tolerance to Embryogenic Cells during Cryopreservation
Source: Int J Mol Sci. 2021 Feb 22;22(4):2174. doi: 10.3390/ijms22042174 (PMC7926411; doi:10.3390/ijms22042174)
Supplement: Supplementary file 1 [file ijms-22-02174-s001.zip › ijms-1099454-supplementary/Lin et al Supplemental data.docx]

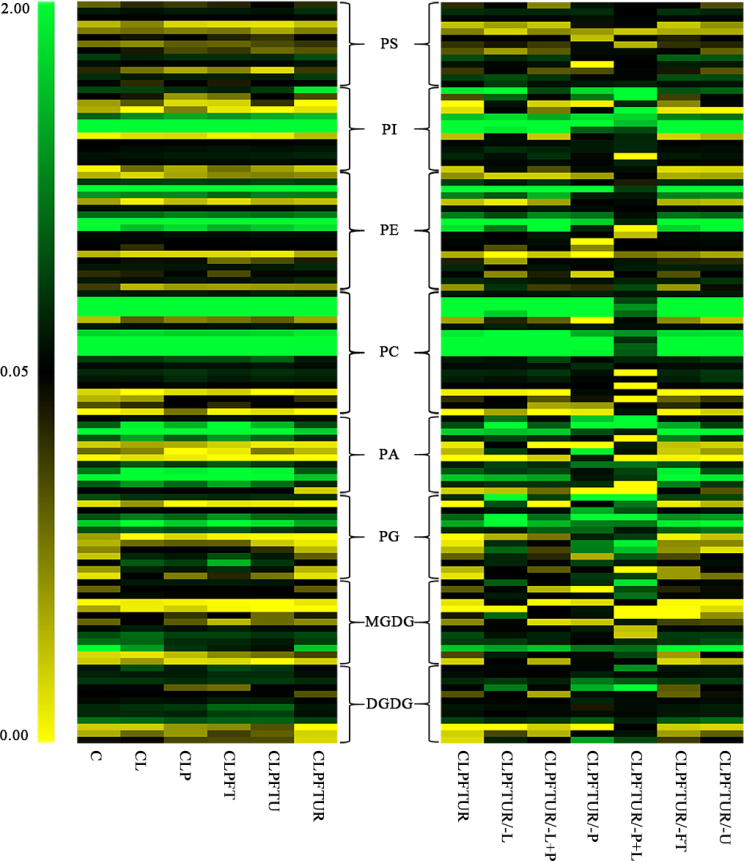


Fig. S1 Effect of cryopreservation processes on the phospholipid profile of embryogenic cells (ECs) of *Magnolia officinalis*. First panel, standard-protocol cryopreservation; second panel, altered-protocol cryopreservation. Each colored bar within a column represents a lipid species in the indicated treatments. The color of each bar represents the level of the corresponding lipid species. Data are expressed as lipid percentage (%). A total of 113 lipid species in the indicated lipid classes are organized using class (as indicated), total acyl carbons (in ascending order within a class) and total double bonds (in ascending order within a class and total acyl carbons).


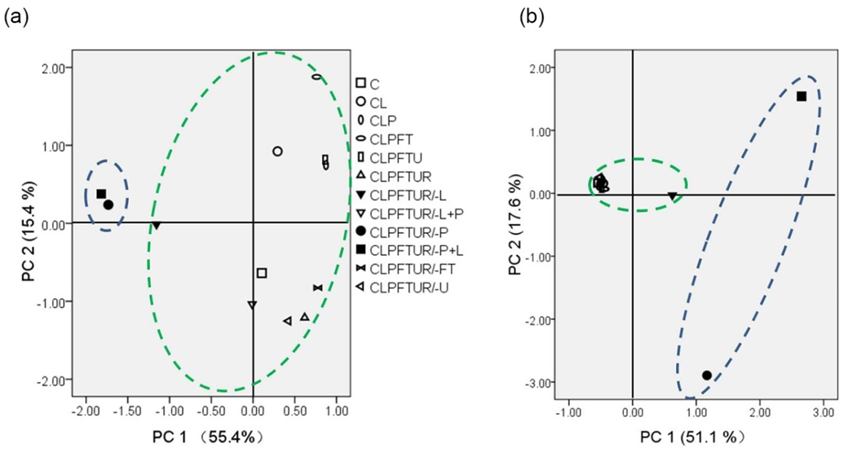


Fig. S2 Principal components analysis (PCA) score plots of the effects of different cryopreservation processes (see Figure 1 legend for notation) on lipid levels, evaluating: (a), lipid content (nmol per mg); and (b), lipid relative content (%). The green group showed fluorescence (Fv/Fm) after treatment, and the purple group did not.


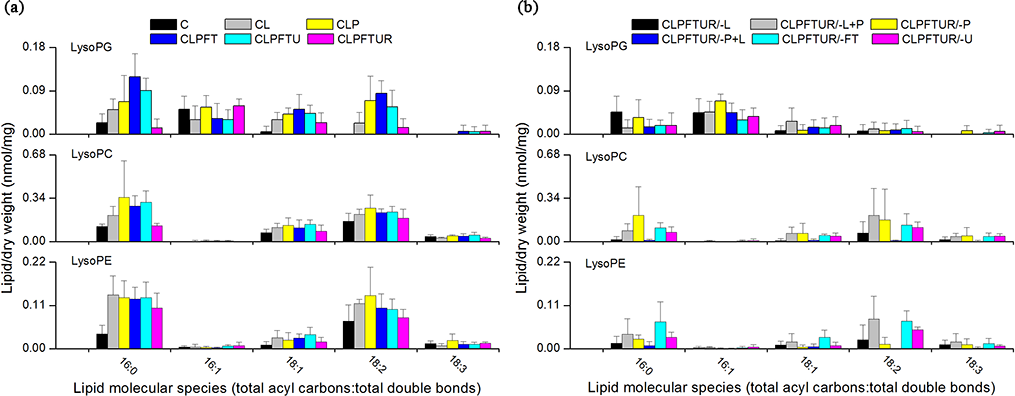


Fig. S3 Changes in levels (nmol/mg) of the molecular species of lysophospholipid molecular species during various cryopreservation treatments of ECs of *Magnolia officinalis*. (a) standard-protocol cryopreservation steps. (b) altered-protocol cryopreservation. Values are mean ± SD (n = 5).


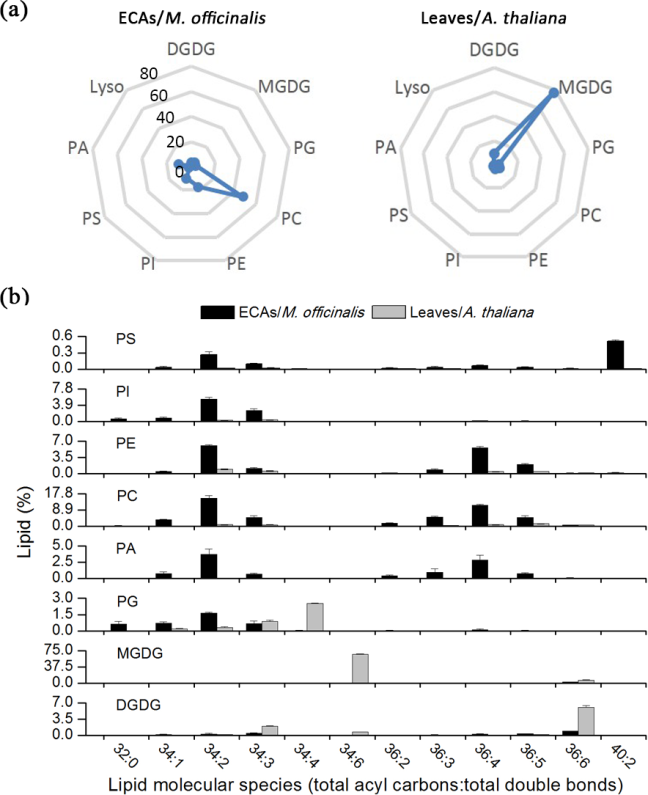


Fig. S4 Composition of lipid class in ECs (*Magnolia officinalis*) and leaves (*Arabidopsis thaliana*). Lipid data for *A.thaliana* from Jia et al. (2016). (a) Percentage of each lipid class in total lipids. (b) Lipid molecular species composition. Values are mean ± SD (n = 4 or 5).

Table S1**.** Loadings of principal components 1 and 2 in analysis of membrane lipid levels (absolute levels) and composition (relative levels).

| Absolute level | | | | |  | Relative level | | | |
| --- | --- | --- | --- | --- | --- | --- | --- | --- | --- |
| Lipid Species | PC 1 loadings | | Lipid species | PC2 loadings |  | Lipid Species | PC 1 loadings | Lipid species | PC2 loadings |
|  | | **Ten highest loading values Ten highest loading values** | | | | | | | |
| PC(36:3) | 0.984 | | PG(36:2) | 0.887 |  | PA(32:0) | 0.998 | PS(36:4) | 0.874 |
| PC(34:1) | 0.979 | | PG(36:5) | 0.809 |  | DGDG(36:1) | 0.995 | PE(40:3) | 0.843 |
| PE(36:3) | 0.978 | | PG(36:3) | 0.807 |  | PG(34:1) | 0.995 | PC(34:4) | 0.648 |
| PC(38:3) | 0.974 | | PG(36:4) | 0.787 |  | PI(32:1) | 0.994 | PE(40:2) | 0.647 |
| PC(36:5) | 0.973 | | MGDG(34:1) | 0.725 |  | PG(32:0) | 0.993 | PE(38:5) | 0.641 |
| PC(32:0) | 0.968 | | PA(34:1) | 0.665 |  | PG(36:1) | 0.990 | PE(38:3) | 0.612 |
| DGDG(34:3) | 0.960 | | DGDG(34:1) | 0.661 |  | PG(36:2) | 0.985 | PI(36:5) | 0.577 |
| PE(34:1) | 0.958 | | DGDG(36:4) | 0.583 |  | PA(34:1) | 0.980 | MGDG(38:4) | 0.574 |
| PE(40:3) | 0.958 | | PG(36:6) | 0.574 |  | PS(44:2) | 0.970 | PE(38:4) | 0.564 |
| PC(34:3) | 0.956 | | DGDG(36:2) | 0.569 |  | PI(36:1) | 0.961 | DGDG(38:3) | 0.562 |
|  | | **Ten lowest loading values Ten lowest loading values** | | | | | | | |
| DGDG(36:1) | -0.023 | | MGDG(38:3) | -0.538 |  | PE(34:1) | -0.893 | PS(34:3) | -0.728 |
| MGDG(34:6) | -0.025 | | PG(34:0) | -0.546 |  | PC(34:2) | -0.903 | PS(36:6) | -0.750 |
| PC(38:6) | -0.197 | | PS(42:2) | -0.553 |  | PE(34:3) | -0.903 | PS(34:1) | -0.792 |
| PI(32:1) | -0.469 | | PS(34:3) | -0.558 |  | PC(34:1) | -0.915 | PC(40:5) | -0.841 |
| PA(34:5) | -0.504 | | PS(40:3) | -0.569 |  | PE(36:4) | -0.917 | PA(34:5) | -0.859 |
| PI(32:3) | -0.526 | | PE(38:5) | -0.576 |  | PC(38:3) | -0.934 | MGDG(34:6) | -0.906 |
| PG(34:4) | -0.542 | | PI(36:1) | -0.634 |  | PC(36:5) | -0.942 | PA(34:6) | -0.914 |
| DGDG(34:4) | -0.559 | | PS(42:4) | -0.643 |  | PE(36:5) | -0.942 | PE(34:4) | -0.930 |
| PG(36:1) | -0.719 | | PS(42:3) | -0.745 |  | PE(34:2) | -0.956 | PC(38:2) | -0.951 |
| PG(32:1) | -0.782 | | MGDG(36:6) | -0.752 |  | PC(36:3) | -0.966 | PS(36:5) | -0.951 |

Table S2 Lipid composition (%) in *Magnolia officinalis* after different cryogenic treatments.

|  | Total PS | Total PI | Total PE | Total PC | Total PA | Total PG | Total MGDG | Total DGDG | Total Lyso |
| --- | --- | --- | --- | --- | --- | --- | --- | --- | --- |
| C | 1.85±0.02^bcde^ | 10.08±1.04^c^ | 17.31±0.67^abc^ | 47.98±3.05^abc^ | 10.31±2.65^cde^ | 3.91±0.36^d^ | 4.51±0.72^ab^ | 3.01±0.46^cd^ | 1.05±0.23^d^ |
| CL | 1.24±0.27^e^ | 9.88±1.33^c^ | 14.83±0.33^c^ | 40.93±2.41^abc^ | 17.80±3.22^ab^ | 6.11±0.73^cd^ | 3.86±0.38^bcd^ | 3.53±0.9^cd^ | 1.82±0.52^cd^ |
| CLP | 1.44±0.28^cde^ | 11.38±1.95^bc^ | 16.22±1.10^abc^ | 45.80±9.08^abc^ | 13.72±6.14^bc^ | 4.75±0.74^d^ | 2.11±0.52^fg^ | 2.78±0.44^cd^ | 1.81±0.85^cd^ |
| CLPFT | 1.44±0.09^cde^ | 9.57±2.47^c^ | 13.01±1.56^cd^ | 40.01±6.12^abc^ | 20.11±7.06^a^ | 7.76±1.55^cd^ | 2.07±0.42^fg^ | 4.20±0.8^bc^ | 1.83±0.31^cd^ |
| CLPFTU | 1.36±0.26^cde^ | 11.02±1.34^c^ | 15.20±1.04^bc^ | 45.96±2.63^abc^ | 14.48±4.83^abc^ | 4.67±0.75^d^ | 1.45±0.13^g^ | 4.14±1.25^bc^ | 1.73±0.29^cd^ |
| CLPFTUR | 2.33±0.2^abc^ | 16.64±2.87^a^ | 18.23±0.89^ab^ | 47.75±1.76^abc^ | 5.77±1.67^e^ | 3.25±0.30^d^ | 3.10±0.42^cdef^ | 1.87±0.28^d^ | 1.05±0.16^d^ |
| CLPFTUR/-L | 3.25±2.03^a^ | 14.89±2.91^ab^ | 11.19±6.21^d^ | 36.69±12.14^cd^ | 9.99±5.5^cde^ | 12.95±11.03^c^ | 4.59±2.37^ab^ | 3.00±1.23^cd^ | 3.45±2.58^c^ |
| CLPFTUR/-L+P | 2.12±0.14^bcde^ | 15.72±1.28^a^ | 18.45±2.29^a^ | 48.40±1.58^ab^ | 5.79±2.90^e^ | 4.21±0.64^d^ | 2.62±0.32^efg^ | 1.51±0.18^d^ | 1.18±0.47^d^ |
| CLPFTUR/-P | 2.31±1.76^abcd^ | 10.02±3.72^c^ | 6.16±2.87^e^ | 27.14±25.1^d^ | 13.45±10.12^bcd^ | 20.23±12.67^b^ | 2.66±1.20^defg^ | 5.95±3.96^ab^ | 12.09±3.9^a^ |
| CLPFTUR/-P+L | 2.26±0.72^abcde^ | 18.04±7.00^a^ | 3.57±1.23^e^ | 7.19±2.55^e^ | 16.92±5.47^abc^ | 32.04±11.93^a^ | 5.69±0.75^a^ | 6.83±3.43^a^ | 7.47±3.23^b^ |
| CLPFTUR/-FT | 2.84±0.47^ab^ | 11.13±2.28^bc^ | 15.94±2.94^abc^ | 43.29±4.97^abc^ | 15.39±8.21^abc^ | 4.71±0.7^d^ | 3.58±0.58^bcde^ | 2.32±0.44^cd^ | 0.81±0.15^d^ |
| CLPFTUR/-U | 2.55±0.53^ab^ | 11.28±2.22^bc^ | 17.26±0.77^abc^ | 49.24±3.1^a^ | 6.55±2.87^de^ | 5.43±1.25^cd^ | 4.16±1.32^bc^ | 2.76±0.92^cd^ | 0.79±0.06^d^ |

Values in the same column marked with differetn letters are significantly different (*p* < 0.05). Values are means ± SD (n = 5).

Table S3 Lipid ratio in ECAs of *Magnolia officinalis* after different cryogenic treatments.

|  | DGDG/MGDG | PC/PE |
| --- | --- | --- |
| C | 0.67±0.07^e^ | 2.77±0.09^abc^ |
| CL | 0.90±0.17^dce^ | 2.76±0.12^abc^ |
| CLP | 1.41±0.48^cd^ | 2.84±0.62^abc^ |
| CLPFT | 2.03±0.08^bc^ | 3.07±0.25^abc^ |
| CLPFTU | 2.85±0.77^a^ | 3.03±0.19^abc^ |
| CLPFTUR | 0.60±0.03^ce^ | 2.62±0.09^bc^ |
| CLPFTUR/-L | 0.69±0.20^ce^ | 3.75±1.35^ab^ |
| CLPFTUR/-L+P | 0.58±0.05^e^ | 2.65±0.32^bc^ |
| CLPFTUR/-P | 2.39±1.39^ab^ | 4.05±2.85^a^ |
| CLPFTUR/-P+L | 1.26±0.73^dc^ | 2.23±1.31^c^ |
| CLPFTUR/-FT | 0.65±0.04^ce^ | 2.76±0.32^abc^ |
| CLPFTUR/-U | 0.66±0.04^ce^ | 2.86±0.23^abc^ |

Values in the same column marked with differetn letters are significantly different (*p* < 0.05). Values are means ± SD (n= 5).

Table S4 Acyl chain length (ACL) of membrane lipids in *Magnolia officinalis* after various cryopreservation process and treatments.

|  | PS | PI | PE | PC | PA | PG | MGDG | DGDG | Total lipid |
| --- | --- | --- | --- | --- | --- | --- | --- | --- | --- |
| C | 39.02±0.30^a^ | 33.96±0.05^ad^ | 35.28±0.05^b^ | 35.05±0.03^bc^ | 34.95±0.03^a^ | 33.77±0.12^cd^ | 35.91±0.06^ab^ | 35.38±0.09^bc^ | 35.04±0.03^a^ |
| CL | 38.61±0.06^ab^ | 33.99±0.09^ad^ | 35.30±0.04^b^ | 35.11±0.07^bc^ | 34.96±0.07^a^ | 34.34±0.1^b^ | 35.79±0.09^abc^ | 35.28±0.16^c^ | 35.03±0.05^a^ |
| CLP | 38.65±0.29^ab^ | 34.04±0.02^abd^ | 35.33±0.02^b^ | 35.12±0.15^bc^ | 35.00±0.03^a^ | 34.27±0.19^b^ | 35.64±0.07^abcd^ | 35.39±0.05^bc^ | 35.05±0.06^a^ |
| CLPFT | 38.69±0.17^a^ | 34.09±0.02^a^ | 35.35±0.6^b^ | 35.19±0.03^bc^ | 35.01±0.05^a^ | 34.59±0.06^a^ | 35.61±0.12^cd^ | 35.33±0.07^c^ | 35.09±0.03^a^ |
| CLPFTU | 38.60±0.36^ab^ | 34.02±0.10^ad^ | 35.30±0.01^b^ | 35.16±0.04^bc^ | 34.98±0.05^a^ | 34.23±0.13^b^ | 35.57±0.09^bcd^ | 35.39±0.04^bc^ | 35.05±0.01^a^ |
| CLPFTUR | 38.84±0.15^a^ | 33.90±0.26^ad^ | 35.44±0.03^b^ | 35.17±0.01^bc^ | 34.97±0.09^a^ | 33.84±0.07^c^ | 35.93±0.07^a^ | 35.35±0.14^c^ | 35.06±0.10^a^ |
| CLPFTUR/-L | 39.27±0.62^a^ | 33.71±0.56^cd^ | 35.82±0.59^b^ | 35.23±0.08^ab^ | 34.50±0.41^b^ | 33.89±0.20^c^ | 35.66±0.48^abcd^ | 35.70±0.36^ab^ | 34.94±0.23^a^ |
| CLPFTUR/-L+P | 38.97±0.14^a^ | 34.04±0.04^ac^ | 35.40±0.05^b^ | 35.26±0.05^ab^ | 34.90±0.16^a^ | 33.88±0.10^c^ | 35.89±0.19^abc^ | 35.48±0.10^abc^ | 35.11±0.03^a^ |
| CLPFTUR/-P | 37.88±1.29^b^ | 33.70±0.38^bd^ | 35.44±0.16^b^ | 34.95±0.28^c^ | 34.10±0.36^c^ | 33.77±0.07^cd^ | 35.42±0.45^d^ | 35.75±0.57^a^ | 34.60±0.29^b^ |
| CLPFTUR/-P+L | 38.78±1.48^a^ | 32.94±0.54^e^ | 38.07±1.95^a^ | 35.49±0.64^a^ | 33.93±0.24^c^ | 33.65±0.24^d^ | 35.04±0.51^e^ | 35.46±0.55^abc^ | 34.19±0.22^b^ |
| CLPFTUR/-FT | 39.16±0.08^a^ | 33.98±0.08^ad^ | 35.43±0.05^b^ | 35.12±0.05^b^ | 35.04±0.06^a^ | 33.83±0.07^c^ | 35.90±0.05^ab^ | 35.29±0.04^c^ | 35.12±0.06^a^ |
| CLPFTUR/-U | 39.23±0.22^a^ | 33.94±0.13^ad^ | 35.49±0.11^b^ | 35.12±0.07^b^ | 34.98±0.06^a^ | 33.90±0.11^c^ | 35.90±0.05^ab^ | 35.40±0.11^bc^ | 35.12±0.07^a^ |

ACL = (Σ[n × mol %lipid)]/100, n is the total number of carbons in the two fatty acid chains of each of glycerolipid molecule. Values in the same column marked with different letters are Significantly different (P < 0.05). Values are mean ± SD (n = 5)

Table S5 Double-bond index (DBI) of membrane lipids in *Magnolia officinalis* after various cryopreservation process and treatments.

|  | PS | PI | PE | PC | PA | PG | MGDG | DGDG | Total lipid |
| --- | --- | --- | --- | --- | --- | --- | --- | --- | --- |
| C | 2.37±0.08^b^ | 2.15±0.03^ab^ | 3.29±0.10^ab^ | 2.95±0.05^ab^ | 2.88±0.06^a^ | 1.72±0.12^c^ | 6.11±0.67b^cd^ | 4.00±0.20^ab^ | 3.04±0.06^a^ |
| CL | 2.34±0.1^b^ | 2.11±0.09^abc^ | 3.37±0.18^ab^ | 2.93±0.05^ab^ | 2.80±0.06^a^ | 2.25±0.13^ab^ | 7.48±0.94^abcd^ | 3.60±0.29^abc^ | 3.04±0.02^a^ |
| CLP | 2.31±0.07^b^ | 2.16±0.03^a^ | 3.32±0.07^ab^ | 2.93±0.14^ab^ | 2.82±0.36^a^ | 2.17±0.17^b^ | 8.27±0.67^ab^ | 3.86±0.13^ab^ | 2.99±0.07^a^ |
| CLPFT | 2.26±0.04^b^ | 2.16±0.03^a^ | 3.35±0.08^ab^ | 2.97±0.03^ab^ | 2.81±0.03^a^ | 2.45±0.05^a^ | 9.53±1.28^a^ | 3.63±0.10^ab^ | 3.03±0.07^a^ |
| CLPFTU | 2.37±0.03^b^ | 2.11±0.10^abc^ | 3.32±0.05^ab^ | 2.98±0.02^ab^ | 2.82±0.05^a^ | 2.09±0.15^b^ | 8.14±1.75^abc^ | 3.69±0.09^ab^ | 2.97±0.04^a^ |
| CLPFTUR | 2.31±0.08^b^ | 1.98±0.26^abc^ | 3.37±0.06^ab^ | 2.94±0.03^ab^ | 2.76±0.11^a^ | 1.62±0.07^c^ | 6.81±0.76^cd^ | 3.94±0.18^ab^ | 2.92±0.07^a^ |
| CLPFTUR/-L | 2.60±0.46^b^ | 1.75±0.55^bc^ | 3.28±0.44^ab^ | 2.95±0.07^ab^ | 2.29±0.40^b^ | 1.40±0.32^d^ | 5.81±1.62^d^ | 3.39±1.09^bc^ | 2.56±0.42^b^ |
| CLPFTUR/-L+P | 2.41±0.05^b^ | 2.19±0.07^a^ | 3.54±0.11^a^ | 3.03±0.04^a^ | 2.74±0.08^a^ | 1.75±0.10^c^ | 6.06±0.54^bcd^ | 4.06±0.33^a^ | 3.00±0.04^a^ |
| CLPFTUR/-P | 3.31±0.93^b^ | 1.72±0.51^cd^ | 3.04±0.17^b^ | 2.65±0.17^c^ | 1.74±0.50^c^ | 1.13±0.17^e^ | 6.72±3.28^bcd^ | 3.76±0.59^ab^ | 2.37±0.38^b^ |
| CLPFTUR/-P+L | 5.46±5.17^a^ | 1.41±0.71^d^ | 3.35±1.16^ab^ | 2.92±0.14^b^ | 1.14±0.29^d^ | 0.99±0.38^e^ | 6.62±3.87^bcd^ | 2.99±1.00^c^ | 1.94±0.26^c^ |
| CLPFTUR/-FT | 2.31±0.02^b^ | 2.11±0.06^abc^ | 3.42±0.04^ab^ | 2.93±0.05^ab^ | 2.89±0.05^a^ | 1.69±0.09^c^ | 6.74±0.27^bcd^ | 4.02±0.09^a^ | 3.00±0.04^a^ |
| CLPFTUR/-U | 2.38±0.06^b^ | 2.10±0.08^abc^ | 3.50±0.07^ab^ | 2.97±0.09^ab^ | 2.87±0.06^a^ | 1.74±0.09^c^ | 6.50±0.58^bcd^ | 4.11±0.28^a^ | 3.05±0.07^a^ |

DBI = (Σ[N × mol %lipid)]/100, N is the total number of double bonds in the two fatty acid chains of each of glycerolipid molecule. Values in the same column marked with different letters are significantly different (P < 0.05). Values are mean ± SD (n = 5)

Table S6 Membrane lipid composition (%), head-group class and lipid ratio in ECAs of *Magnolia officinalis* and leaves of *Arabidopsis thaliana*. Lipid data for *A.thanalia* from Jia et al. (2016).

|  | Tissue/Plant Species | |
| --- | --- | --- |
| Lipid Class | ECAs/*M.officinali* | Leaves/*A.thaliana* |
| PS | 1.85±0.03^a^ | 0.09±0.04^b^ |
| PI | 10.08±1.04^a^ | 0.73±0.15^b^ |
| PE | 17.31±0.67^a^ | 3.04±0.31^b^ |
| PC | 47.98±3.05^a^ | 4.91±0.55^b^ |
| PA | 10.31±2.65^a^ | 0.02±0.02^b^ |
| PG | 3.91±0.36^a^ | 3.92±0.16^a^ |
| MGDG | 4.51±0.72^b^ | 77.55±1.22^a^ |
| DGDG | 3.01±0.46^b^ | 9.70±0.54^a^ |
| Lyso | 1.05±0.23^a^ | 0.03±0.003^a^ |
|  | Lipid ratio | |
| DGDG/MGDG | 0.67±0.07^a^ | 0.13±0.01^b^ |
| PC/PE | 2.77±0.09^a^ | 1.62±0.07^b^ |
| Galactolipids/Phospholipids | 0.08±0.01^b^ | 6.90±0.63^a^ |

Values in the same column marked with differetn letters are significantly different (*p* < 0.05). Values are means ± SD (n= 4 or 5).
